# Comparative Analysis of Erosive Wear Behaviour of Epoxy, Polyester and Vinyl Esters Based Thermosetting Polymer Composites for Human Prosthetic Applications Using Taguchi Design

**DOI:** 10.3390/polym13203607

**Published:** 2021-10-19

**Authors:** Jeetendra Mohan Khare, Sanjeev Dahiya, Brijesh Gangil, Lalit Ranakoti, Shubham Sharma, Muhammad Roslim Muhammad Huzaifah, Rushdan Ahmad Ilyas, Shashi Prakash Dwivedi, Somnath Chattopadhyaya, Huseyin Cagan Kilinc, Changhe Li

**Affiliations:** 1 School of Engineering & Technology, University of Technology, Rajasthan, Jaipur 303903, India; jmkhare2010@gmail.com (J.M.K.); dahiyasanjeev764@gmail.com (S.D.); 2 Department of Mechanical Engineering, H.N.B. Garhwal University, Garhwal, Srinagar 246174, India; brijeshgangil7@gmail.com; 3 Department of Mechanical Engineering, National Institute of Technology (NIT Uttarakhand), Srinagar 246174, India; lalitranakoti9000@gmail.com; 4 Department of Mechanical Engineering, IK Gujral Punjab Technical University, Main Campus, Kapurthala 144603, India; 5 Department of Crop Science, Faculty of Agricultural Science and Forestry, Universiti Putra Malaysia Bintulu Campus, Bintulu 97000, Malaysia; 6 School of Chemical and Energy Engineering, Faculty of Engineering, Universiti Teknologi Malaysia, Johor Bahru 81310, Malaysia; ahmadilyas@utm.my; 7 Centre for Advanced Composite Materials, Universiti Teknologi Malaysia, Johor Bahru 81310, Malaysia; 8 G.L. Bajaj Institute of Technology & Management, Greater Noida 201310, India; spdglb@gmail.com; 9 Department of Mechanical Engineering, Indian Institute of Technology (ISM) Dhanbad, Dhanbad 826004, India; somnathchattopadhyaya@iitism.ac.in; 10 Civil Engineering Department, Istanbul Esenyurt University, Istanbul 34510, Turkey; huseyincagankilinc@esenyurt.edu.tr; 11 School of Mechanical and Automotive Engineering, Qingdao University of Technology, Qingdao 266520, China; sy_lichanghe@163.com

**Keywords:** natural and synthetic fibers, thermosetting polymers, L_16_ orthogonal array, Taguchi method, erosion mechanism, SEM analysis, prosthetic applications

## Abstract

In polymer composites, synthetic fibers are primarily used as a chief reinforcing material, with a wide range of applications, and are therefore essential to study. In the present work, we carried out the erosive wear of natural and synthetic fiber-based polymer composites. Glass fiber with jute and *Grewia optiva* fiber was reinforced in three different polymer resins: epoxy, vinyl ester and polyester. The hand lay-up method was used for the fabrication of composites. L_16_ orthogonal array of Taguchi method used to identify the most significant parameters (impact velocity, fiber content, and impingement angle) in the analysis of erosive wear. ANOVA analysis revealed that the most influential parameter was in the erosive wear analysis was impact velocity followed by fiber content and impingement angle. It was also observed that polyester-based composites exhibited the highest erosive wear followed by vinyl ester-based composites, and epoxy-based composites showed the lowest erosive wear. From the present study, it may be attributed that the low hardness of the polyester resulting in low resistance against the impact of erodent particles. The SEM analysis furthermore illustrates the mechanism took place during the wear examination of all three types of composites at highest fiber loading. A thorough assessment uncovers brittle fractures in certain regions, implying that a marginal amount of impact forces was also acting on the fabricated samples. The developed fiber-reinforced polymer sandwich composite materials possess excellent biocompatibility, desirable promising properties for prosthetic, orthopaedic, and bone-fracture implant uses.

## 1. Introduction

Fiber-reinforced polymer composite materials are the modern materials applied in various applications such as automobile interiors, construction articles, transportation materials, packaging, and household ware [1,2,3]. The prerequisite necessary for all the aforementioned applications is good strength, stiffness, durability, flexibility, etc., which a composite does exhibits as reported by several literatures in the past decades [4,5,6]. Synthetic fiber-reinforced polymer composites have several advantages such as extremely high strength comparable to metals but have several demerits such as high carbon release in the environment, non-disposable leading to soil degradation, high cost, etc. [7]. On the other hand, natural fiber reinforced polymer composites exhibit quite remarkable mechanical properties but far lower than synthetic counterparts [8,9,10]. To optimize parameters such as strength, cost, etc., and minimize the hazardous environmental effects, hybridization of natural and synthetic fiber has been performed. For instance, the hybrid composite was prepared by incorporating Cocos nucifera and Lufa cylindrical fiber at a weight fraction of 30% in the mix of MEKP and cobalt naphthenate and reported that mechanical properties improved by 31%. It was also reported that changing the fiber ratio in the composition led to alteration of property from ductile to brittle [11]. The addition of cellulosic fiber (banana, abaca, jute, and hemp, wood) in glass fiber reinforced polymer hybrid composite yields higher mechanical strength than composite containing only glass fiber [12,13,14]. Analysis of the hybrid composite of synthetic-synthetic fiber [15] and plant-animal fiber [16,17] have also been carried out and reported improved results regarding mechanical properties. Kevlar-kenaf fiber reinforced polymer hybrid composite was fabricated to investigate mechanical properties [18,19] and reported that significant enhancement can be obtained in the impact properties at 40 wt% of fiber reinforcement. Automobile door panels and several automotive parts are under consideration to be manufactured from the hybrid composite of hemp and kenaf fiber [20]. 

Glass fiber is one of the strongest known fiber primarily used as a chief reinforcing material in the polymer composite with a wide range of applications. However, due to its high cost, several natural fibers were reinforced to make the composite economic. In addition, glass fiber is highly brittle and, therefore, mostly reinforced with ductile natural fiber to reduce the brittleness of hybrid polymer composite [21]. In recent times, various natural fibers have been reinforced with glass fiber to enhance the mechanical properties and wear resistance of glass fiber polymer composite. For instance, reinforcement of fibers such as bamboo, sisal, and wood in glass fiber composite enhanced tensile, flexural, and impact properties of hybrid composite [22,23,24]. Silica nanoparticles filled glass fiber reinforced epoxy composites yield five times higher fatigue strength than virgin glass fiber composite [25]. Fracture strength, interlaminar shear strength, flexural strength, and impact strength were improved by incorporating flax, basalt, and jute fiber in glass fiber reinforced polymer composite [26,27,28,29]. It has been observed that the weightage of natural fiber was kept below the weightage of glass fiber in the hybrid composite to obtain optimum mechanical strength [30,31,32]. The mechanical and tribological properties of a hybrid composite comprising more than two fibers, primarily two natural and one glass fiber, were also investigated. In this regard, glass fiber/sisal fiber/chitosan reinforced polymer sandwich were fabricated for orthopaedic fracture application and reported impressive wear resistance and modulus [33]. Hybridization of 10% jute and 10% tea leaf fiber in glass yielded mechanical strength of such value that can even replace virgin glass fiber composite [34]. Reinforcing jute, sisal, kenaf, or combining the two in glass fiber reinforced polymer composites also improves the mechanical properties. [35,36]. 

Material erosion caused by hard particles is one of several types of material degradation categorized as wear. Polymers composites also work under different working conditions, requiring an analysis of their wear activities before they are located in a real environment. Several studies have been reported in the past for the investigation of erosive wear of polymer composite containing different types of fiber and fillers [37]. For instance, glass fiber reinforced polymer composite filled with micro silica and zinc oxide was fabricated via vacuum-assisted method and investigated for erosive wear behavior at a different impingent angle ranging from 20° to 90° [38]. It was reported that silica fumes enhanced the composite’s erosive wear resistance, while zinc oxide promoted erosive wear.

**Figure 1:**
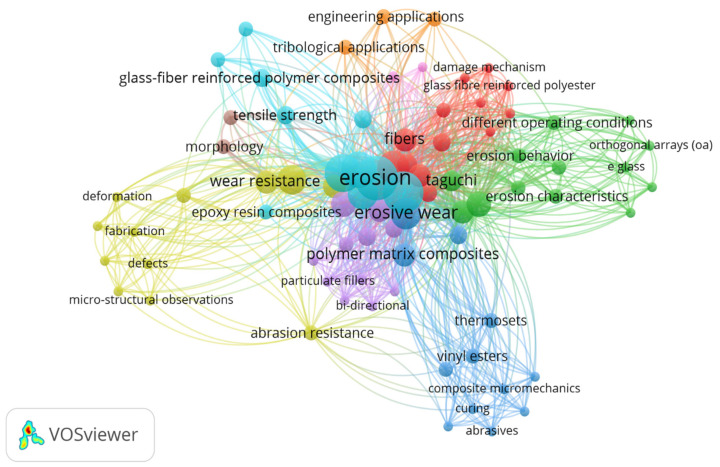
Bibliometrics visualisation overview of technology-based progressions on the erosion-behaviour of composites obtained from the various thermoset-resins (epoxy, polyester, and vinyl-ester) for a multitude of scenarios.

Moreover, increasing the impingement angle and size of erodent particles increased the erosive wear. The incorporation of marble dust in glass fiber reinforced polymer composite reduces erosive wear of the fabricated composite, as marble dust increases the hardness and stiffness of the composite surface [39]. Diversifying the process parameters and input variables such as matrix type, filling material, and manufacturing method can reduce the erosive wear of the manufactured composites. Calcium carbonate (CaCO_3_), barium sulphate (BaSO_4_), and tungsten carbide (WC) filled glass fiber reinforced PA/ABS composite prepared by injection molding exhibited relatively lower erosive wear at higher impingement angle, i.e., 90° or 75° [40,41,42]. However, the incorporation of CaCO_3_ and BaSO_4_ promoted ductile and semi ductile erosive wear while WC promoted brittle wear of the composite. Treatment of fiber by chemical agents plays a crucial role in enhancing the resistance of composite against erosive wear. Benzoylated treated areca sheath fiber reinforced polyvinyl chloride exhibited lower erosive wear than untreated areca sheath fiber-reinforced composite [43]. In addition, fiber treatment encourages good bonding between fiber and matrix resulted in low erosive wear efficiency. The erosive wear of carbon and glass fibre reinforced composites demonstrated that they could attain excellent erosive wear resistance without using any ceramic filler; however, this was not the case with natural fibre reinforced composites. [44]. It is interesting to observe from the literature that synthetic fiber reinforced composites exhibited lower erosive wear than natural fiber-reinforced polymer composite. Jute fiber has excellent strength, good UV protection, low thermal conduction and attractive anti-static properties which qualifies it a good reinforcing material. In addition, low cost *Grewia optiva* fiber, low density, easily available in the Himalayan region contains high amount of pectin (jelling and thickening agent) which is advantageous in making good bonding with polymeric chain and can be useful in a way of making cheaper and lighter prosthesis with good mechanical and tribological properties. As discussed in the literature, research on the erosive wear of composites combining both natural and synthetic fibers has been limited. As a result, the current research focuses on the hybridization of synthetic and natural fibres and compares the erosive wear of composites comprising three different resins: epoxy, polyester, and vinyl ester, using the Taguchi methodology as shown in the Figure 1.

## 2. Experimentation

### 2.1. Materials

Bisphenol resin and epichlorohydrin were purchased from HEXION Specialty Chemicals Pvt. Ltd. Karnataka and mixed in the ratio of 5:1 for the preparation of epoxy (Epikote Resin 828), having good chemical resistance, internal adhesion, and appropriate wetting pigment. Esterification of epoxy with unsaturated mono carboxylic acid purchased from Amtech Ester Pvt. Ltd. Delhi was performed to prepare vinyl ester and dissolve the reactant in the solution of the solution styrene to provide stability to the prepared vinyl ester. Dibasic organic acids with polyhydric alcohols were purchased from Yes composites India Ltd. New Delhi and mixed in the appropriate ratio for the preparation of polyester.

**Figure 2:**
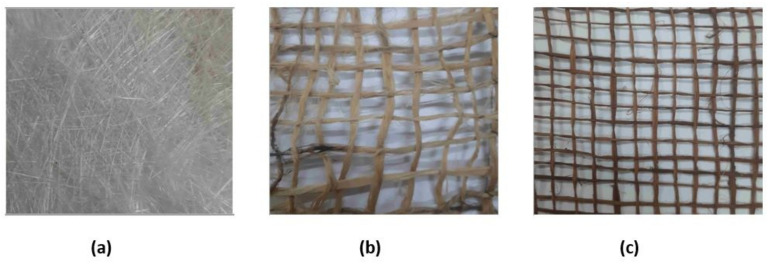
Showing (**a**) Glass fiber, (**b**) Jute fiber and (**c**) *Grewia optiva* fiber.

Strand of chopped glass fiber having good strength and high insulating properties were procured from Yes composite Ltd. Natural fibers, i.e., jute and *Grewia optiva* were purchased in the form of bi-directional mat locally from the Uttarakhand Bamboo Board (India). These fibers were treated from NaOH solution with 8% concentration and then washed in distilled to remove dirt and dust present on the surface of fibers. Fibers used in the present investigation are shown in Figure 2.

### 2.2. Methods

**Figure 3:**
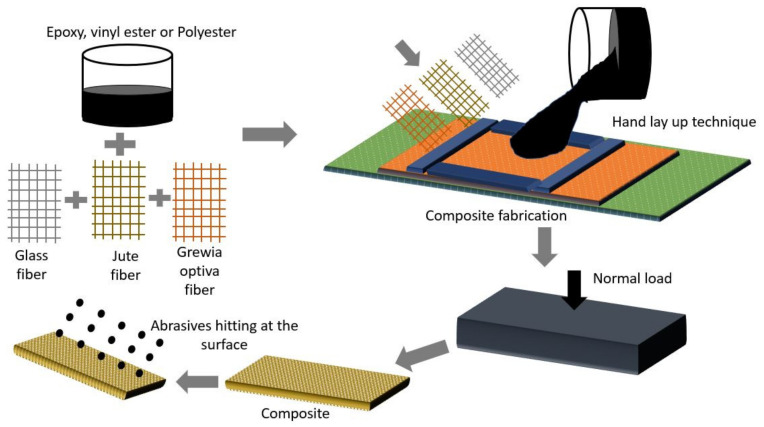
Process of fabrication of composite.

**Figure 4:**
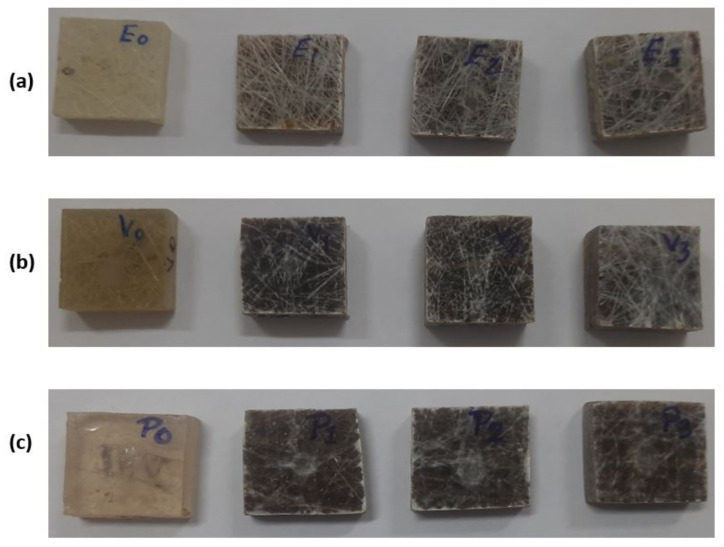
(**a**) Epoxy based, (**b**) vinyl ester based and (**c**) poly ester-based sample for erosive wear test.

The composite samples were made using the hand lay-up approach, as shown in Figure 3. Glass plates measuring 500 × 300 × 4 mm^3^ were utilized as molding plates for composite manufacturing. Double-sided tape was used on all sides of the molding plate to achieve the desired thickness and secure the side bidding of the fabricated composite. Silicon spray was used over the molding plates to avoid the sticking of the sample with the plates. Firstly the resin was poured over the molding plates and evenly dispersed with the help of a steel roller, after which the natural and synthetic fibre mat of known percentage were placed one by one over the resin. Subsequently, the remaining resin was spread evenly over the mat with the help of a roller. Finally, a 15 kg load was held above the sample and left to cure. The composite sample was taken out of the mould and cut to the appropriate dimension for erosive wear characterization after 24 h of curing, as shown in Figure 4. Samples fabricated by varying fiber weightage are illustrated in Table 1. 

### 2.3. Erosive Wear Analysis

**Figure 5:**
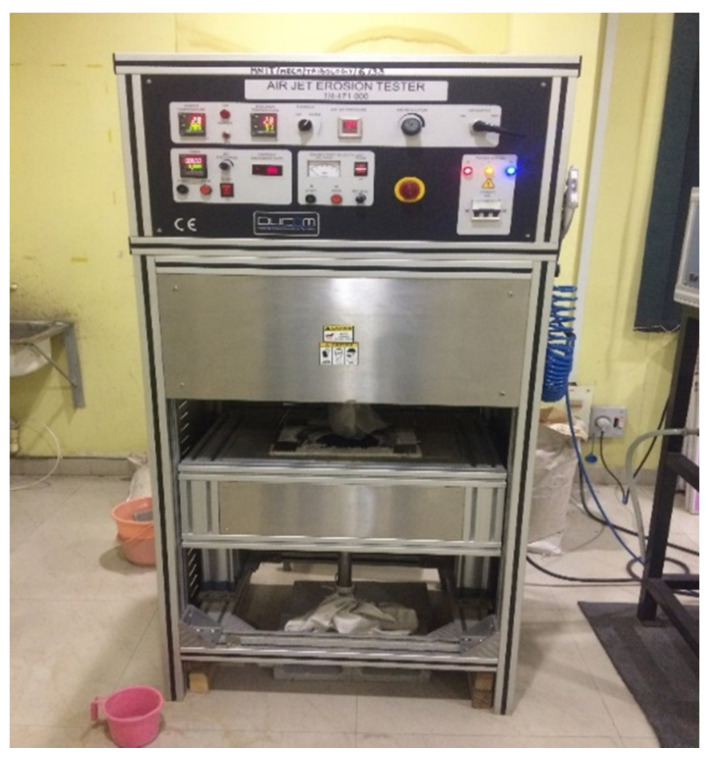
Air jet erosion test machine set up.

The analysis of erosive wear for the fabricated samples was carried out as per ASTM-G 76 standard of size 30 × 30 × 5 mm^3^ on air jet erosion tester supplied by DUCOM, India as shown in Figure 5. The erodent particles used in the experiment were silica of size varying from 100 to 250 μm. Silica particles were forced to impinge at the surface of the sample through a tungsten carbide nozzle for 15 min at different experimental conditions. After completing the test, the surface of the samples was cleaned by using acetone, and an electronic weighing machine measured its weight.

### 2.4. Taguchi Experiment Design

Various control factors influence the erosive wear, such as the size of the erosive particle, velocity of impact, angle of impingement, filler content, concentration, etc. In this present investigation, erosive wear is assessed by evaluating three control factors, each having four levels as tabulated in Table 2. 

Considering Table 2, if all the experiments are to be performed, it will be 81 numbers for three control factors and four levels. It will become cumbersome and laborious. Moreover, a lot of time and energy will be required, which makes it a costly deal. Alternatively, the Taguchi approach can be applied, which uses an orthogonal array to break down the 81 numbers in just a handful of experiments offering enough control factors as provided by 81 experiments. Here, L_16_ orthogonal array has been constructed as shown in Table 3 for the investigation in the Minitab to analyse erosive wear. Moreover, signal to noise (S/N) ratio was analysed using lower the better characteristics as per equation 1to examine the erosive wear of the composite samples. 

Lower-the-better characteristic:

(1) $$S/N\;\mathrm{ratio}=-10log\frac{1}{n}\left(\sum_{i=1}^{n}y_{i}\right)$$

Here, y is erosive wear and *n* is number of experiments.

#### Literature-Based on Erosive Wear Analysis of Polymer Composites Using Taguchi Approach

The erosive wear analysis of fiber/filler reinforced polymer composites has been carried out recently at different impact velocities, filler content, impingement angle, erodent size, etc., as shown in Table 4. Several types of orthogonal array have been used to examine the effect of different control factors on the erosive wear of composite. Table 4 suggests that erosive wear gets significantly affected by altering the controlling factors. Initially, orthogonal arrays were used only for composite containing epoxy or polyester based composites. But limited or none of the studies were done on vinyl ester-based composites. Furthermore, the effect of three fibers in a composite with different resins has rarely been investigated.

## 3. Results and Discussions 

### 3.1. Mechanical Properties 

The properties shown in Table 5 are the average of 3 readings taken for each sample which has taken from previous research [55]. When the tensile strength of composites was compared, it was discovered that epoxy-based composites had the maximum tensile strength, 72 MPa, at 15 wt% loading of jute and *Grewia optiva* fibre. However, in the case of flexural strength, the vinyl ester based composites outperformed both epoxy and polyester-based composites with the highest value of 48 MPa at 5 wt% loading of both jute and *Grewia optiva* fiber. The impact and hardness values were found to be higher for epoxy-based composites. The properties depicted in the Table 5 shows that the fiber reinforcement in epoxy is more advantageous than reinforcement in vinyl ester and polyester due to higher overall enhancement of mechanical properties. The reduction of the mechanical property in viny ester and polyester composites can be related to the photochemical degradation, plasticizing effect, and weak interfacial adhesion, weakening the interface between the matrix and fillers. On the other hand, epoxy-based composites accomplish efficient mechanical interlocking between the fibre and epoxy, resulting in good stress transfer from the epoxy to the fiber [56,57].

### 3.2. Taguchi Analysis of Erosive Wear

On the prepared composites, three control factors and four levels were investigated using the L_16_ orthogonal array. The analysis of several combinations of control factors was performed using Minitab 15. Erosive wear rates of different resin-based composite and their corresponding S/N ratio have been presented in Table 6. Furthermore, the effect of control factor on the erosive wear with the respective ranking is tabulated in Table 6. Observations revealed that the control factor which influenced erosive the most was found to be impact velocity. The effect of fiber content on erosive wear was low as compared to impact velocity but quite considerable. Impingement angle has the least effect on erosive wear among all the three control factors. Further, a graph of control factors at different levels is shown in Figure 4. For epoxy-based composites, it can be concluded from Table 6 that erosive wear increases with the increase in impact velocity and is found to be minimum at an Impact velocity of 30 m/s, 15 wt% fiber reinforcement and impingement angle of 90° whereas maximum erosive wear was obtained at 60 m/s 0 wt% fiber reinforcement and impingement angle of 90°. This indicates that the addition of fiber in epoxy does not necessarily influence the erosive wear but also the impingement angle. When the erodent particles hit the composite surface, the epoxy material is first contacted and then the reinforcement after the erosion. The Rockwell hardness of epoxy is relatively high, with an outstanding value of 80 number, which is capable of bearing the impact of erodent particle at such high velocity. The respective S/N ratio at 3rd run and 13th run erosive wear is also the evidence for minimum and maximum erosive wear. 

It can also be observed from Table 6 that the erosive wear of vinyl ester composites among the 16 runs was found to be minimum at 7th run maximum at 14th run, which is at an impact velocity of 40 m/s, fiber weightage of 10 wt%, 90° impingement angle and impact velocity of 60 m/s, fiber weightage of 0 wt%, impingement angle of 75°. The magnitude of erosive wear was approximately similar to epoxy-based composites but 0.7% higher in magnitude. Interestingly, the most increased erosive wear occurs at 75° impingement angle and 0 wt%. This shows that fiber inclusion in the vinyl ester composites reduces the erosive wear, and impingement angles from 75° to 90° showed the same erosive wear. Moreover, the upsurge in impact velocity increases the erosive wear of the vinyl ester composite. 

Erosive wear for polyester-based composites was found to be higher as compared to both epoxy and vinyl ester-based composites. It was observed that minimum and maximum erosive wear were obtained at an impact velocity of 30 m/s, fiber reinforcement of 0 wt%, 75° impingement angle (3rd run) and impact velocity of 60 m/s, fiber reinforcement of 0 wt% and impingement angle of 90° (13th run), respectively. The reason of higher wear may be attributed to the low hardness of polyester resulting in low resistance against the impact of erodent particles [58].

**Figure 6:**
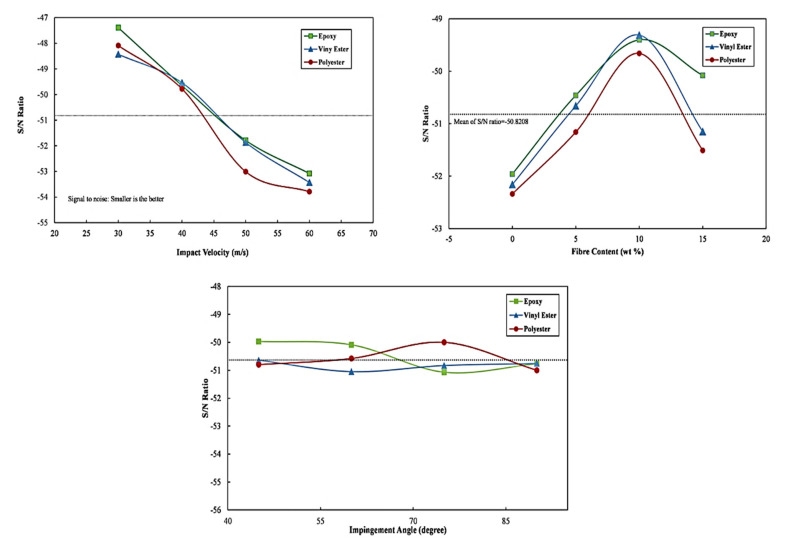
Mean of S/N ratio for epoxy (ep), vinyl ester (ve) and polyester (pe) based composites.

The analysis of results as shown in Figure 6 concludes that the combination at an impact velocity of 30 m/s, fiber content of 10 wt% and impingement angles of 45° yields the lowest erosive wear in epoxy-based composites. For the vinyl ester composites, the lowest wear rate was obtained for the combination at impact velocity of 30 m/s and fiber content of 10 wt% and impingement angles of 45°. Additionally, in the case of polyester-based composite, the lowest erosive wear was obtained at 30 m/s, fiber content of 10 wt% and impingement angles of 45°. Furthermore, as described in Table 7, the order of effectiveness of control factors is impact velocity (1st), fiber content (2nd), and impingement angle (3rd).

#### Analysis of Variance 

**Figure 7:**
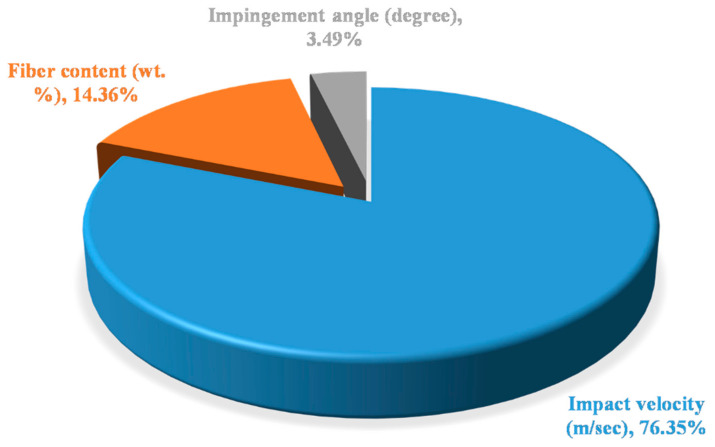
Contribution chart of factors for epoxy-based composites.

Analysis of variance (ANOVA) was used for the analysis of the statistical significance of control factors at 95% confidence level. The *p*-value of control factors or epoxy-based composites is shown in Table 8 which shows the degree of significance of the control factor on erosive wear. It was observed that the value of *p* for impact velocity is 0.001 which is lower than assumed *p*-value, i.e., 0.05 and is the most significant factor for the analysis of erosive wear. The next significant factor was fiber content with the *p*-value of 0.046. Impingement angle was observed to be least significant factor as per the ANOVA Table 8. The contribution of all the factors for epoxy-based composites with the highest contribution of impact velocity has a value of 76.35%, followed by fiber content with the contribution factor of 14.36% and the least contribution of impingement angle with 3.49% is shown in Figure 7. 

**Figure 8:**
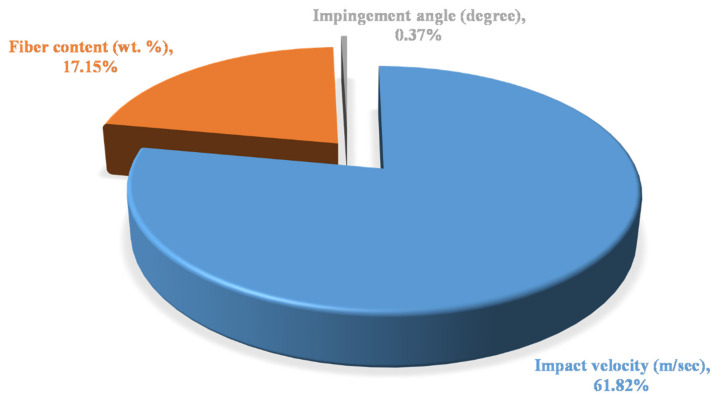
Contribution chart of factors for vinyl ester-based composites.

The *p*-value of control factors or vinyl ester-based composites is shown in Table 9. It was observed that the value of *p* for impact velocity is 0.0031, which is lower than the assumed *p*-value, i.e., 0.05, and is the most significant factor for the analysis of erosive wear. The next significant factor was fiber content with the *p*-value of 0.275. Impingement angle was observed to be the least significant factor as per the ANOVA Table 9. The contribution of all the factors for vinyl ester-based composites with the highest contribution of impact velocity has a value of 61.82%, followed by fiber content with the contribution factor of 17.15% and the least contribution of impingement angle with 0.37% is shown in Figure 8.

**Figure 9:**
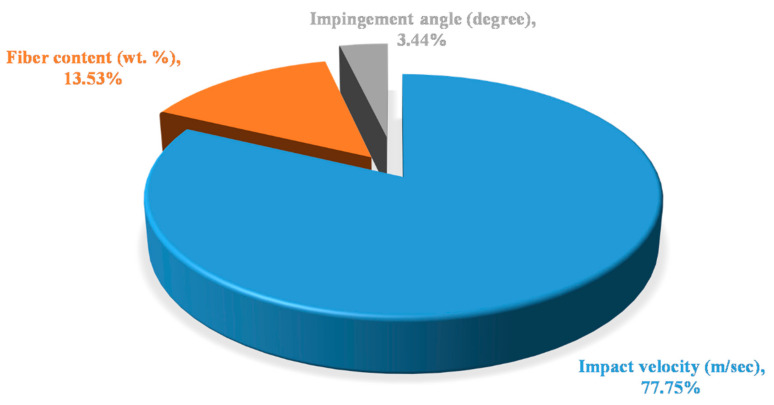
Contribution chart of factors for polyester based composites.

The *p*-value of control factors or polyester-based composites is shown in Table 10. It was observed that the value of *p* for impact velocity is 0.001, which is lower than the assumed *p*-value, i.e., 0.05, and is the most significant factor for the analysis of erosive wear. The next significant factor was fiber content with a *p*-value of 0.043. Impingement angle was observed to be least significant factor as per the ANOVA Table 10. The contribution of all the factors for epoxy-based composites with the highest contribution of impact velocity has a value of 77.75%, followed by fiber content with the contribution factor of 13.53% and least contribution of impingement angle with 3.44% is shown in Figure 9.

### 3.3. Morphological Analysis

**Figure 10:**
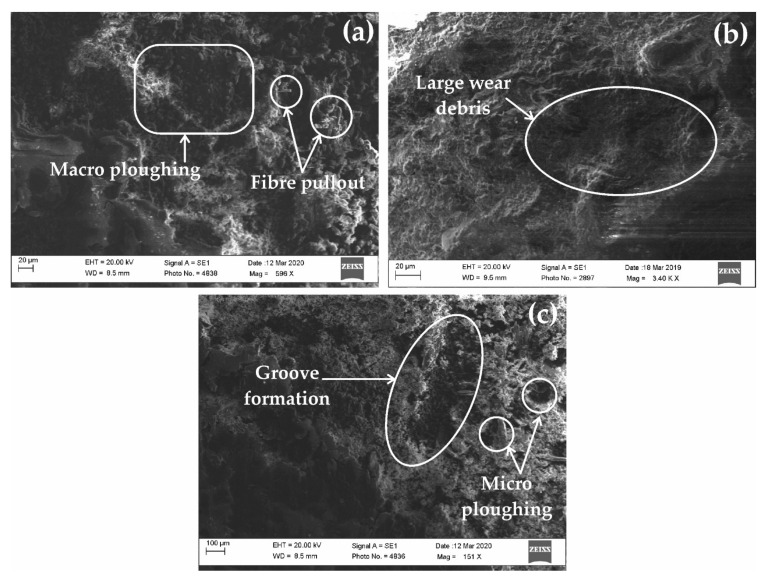
SEM images at highest fiber loading (**a**) epoxy based composite, (**b**) vinyl ester and (**c**) polyester based composite.

The Figure 10 illustrates the mechanism took place during the wear examination of all three types of composites at highest fiber loading. Epoxy based composites as shown in Figure 10a exhibited fiber pull out leading to exposure of fibers with the wear surface [56,57,58,59,60,61,62]. The patches of ploughing at macro level have also been observed which may be considered as the major factor in the wear of the composite. However, the overall wear of epoxy-based composites reduced by the interaction of fiber with the mating surface took place by fiber pull out. It is to be noted that addition of jute and grewia fibers in the glass fiber-epoxy composites increases the erosive wear till 10 wt% of loading. However, on increasing the natural fiber weightage beyond 10% loading, the erosive wear behaviour reduces significantly. Large wear debris was spotted in vinyl ester-based composites (Figure 10b). These wear debris formed due to the detachment of sub polymeric material from base material due to low van der wall forces. Here, the exposure of fiber is negligible which somehow can be linked to the comparative higher wear than epoxy-based composites. In case of polyester composites (Figure 10c). The bonding between the natural fiber and ester group of vinyl ester and polyester matrix is comparatively low as compared to epoxy-natural fiber bonding which triggered more fiber detachment. Interestingly, at higher natural fiber loading (15 wt% jute and grewia), the improvement in the erosive wear was not significant as obtained in the study. Apparently, the erosive wear examination revealed that main wear mechanism responsible for material removal is groove formation and micro-ploughing [63,64,65]. A close examination also discloses brittle fractures at some parts, which shows that a small amount of impact forces was also acting on the samples. 

**Figure 11:**
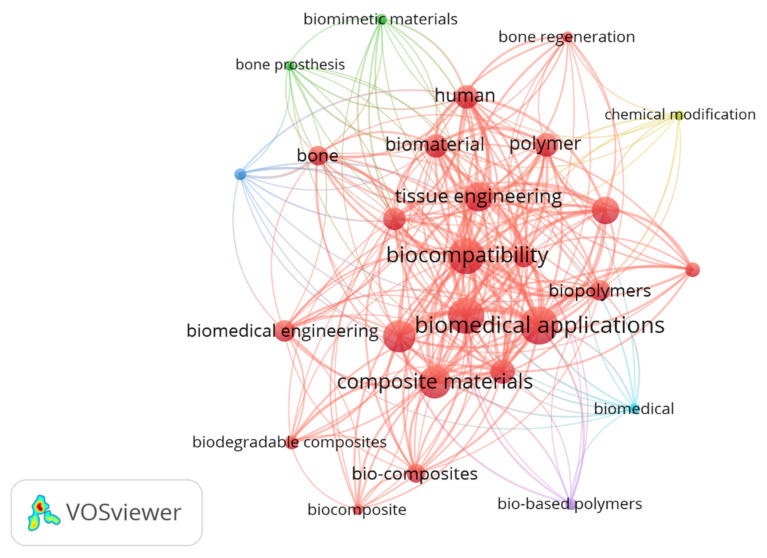
Bibliometric assessment on the applications of natural fiber/synthetic fiber reinforced thermosetting polymeric composites for biomedical and human prosthetic applications.

The scientometric analysis emphasizing the highlights especially on several of the most notable biocomposite material findings available in the literary works with a prominence on the biocompatibility, and material characteristics of biocomposites for artificial-limp/prosthetic applications as displayed in the Figure 11. Novel bio-based composites have mostly been evolved in response with a burgeoning consumption for eco-friendly sustainable materials and the willingness to minimise the expenditure with conventional fibres reinforced fossil-fuel derived composites. Investigators had already focused primarily on biocomposites, which are constituted of naturally or synthetic resins derived from the natural fibre-reinforcements. Natural fibres have significant upsides as they are a light-density material which tends to produce comparatively light-weight composites with slightly elevated unique characteristics [63,64,65,66,67,68,69,70]. Such filaments now provide substantial savings, efficient use of resources, and processability, and seems to be a profoundly renewable energy source, aiding to curtail dependence on international as well as household petroleum products. In the current context, self-sustaining environmentally sound approaches to traditional materials, explicitly glass-fibres, are now being deemed to be used in the lamination of artificial-limbs or prostheses connectors.

Utilizing polymer’s higher-thermal conversion power-density, delivery methods that rely on these compounds were being used in photo-thermal therapeutic-treatment [71]. The investigators had summarized the findings, emphasized that, whilst area is still very much in beginning phases, conjugated-polymeric/poly-electrolyte interfacial-interactions have vast potential for healthcare applications [71].

Throughout this perspective, one of the most important aspects of sustainable-development is being utilised the organic-matter (biomass), and its compounds as a predecessor of carbon-materials [72]. A concise summarization of current developments in the synthesizing approach of self-sustaining carbon-compounds and their promising implications has been investigated. This report discusses fundamental observations and crucial recommendations for the eventual development of green carbon-materials and their burgeoning usage in catalytic and healthcare [72].

Metabolism fingerprinting of biological fluids record a wide range of disorders, and urinary-detecting, in particularly, provides ideal non-invasiveness towards upcoming diagnoses [73]. Owing with a restricted bio-markers and higher sampling intricacy, existing urinary identification provides major shortcomings and necessitates use of sophisticated materials to extract biomolecular data. Polymer@Ag generated urinary meta-bolic fingerprinting (UMFs) by LDI-MS within seconds employing approximately One Litre of urinary without enriching or purifying [73].

Selective target, trans-membrane distribution, transport, and stimulation responsiveness could all be integrated within peptide-based theranostic nanostructures [74]. Throughout the article, the researchers had discussed generalized principles for synthesising peptide-based therapeutic, and diagnostic nano-materials, with a focus on performance, design, and numerous bio-medical possibilities, and they have illustrated their significant development over the last five years [74].

The developed microchip identifies tiny metabolite-molecules in human plasma rapidly, sensitively, and preferentially without enriching or purifying [75]. On-chip plasma fingerprinting enable further distinction among women having ovarian/colorectal cancer & control-subjects, and also therapeutic assessment for significant medical surveillance. The research explored the use of laser-de-sorption or ionisation mass-spectroscopy in huge therapeutic medicinal towards vitro-testing [75].

The co-workers have proposed synthesized Palladium–Gold alloys using mass-spectroscopy-based metabolite fingerprint, and assessment throughout the diagnostic and radiation treatment of medullo-blastoma [76]. Deep learning has been employed to identify medullo-blastoma individuals, whereas radiation therapy has been observed and an initial array of plasma metabolites-biomarkers was discovered exhibiting progressive alterations [76].

## 4. Conclusions

Natural fiber/synthetic fiber reinforced polymeric composites have been successfully fabricated with three different polymer resins by hand lay-up process for the analysis of erosive wear behavior. Taguchi technique L_16_ orthogonal array was used to optimize the number of experiments. The obtained results from the investigation revealed that impact velocity is the most significant control factor in the analysis of erosive wear. The second most considerable control was fiber content, followed by impingement angle for all the composites. Polyester-based composites exhibited the highest erosive wear among all the composites, followed by vinyl ester. Epoxy-based composites showed the least erosive wear among all. The contribution charts found that impact velocity has the highest contribution in polyester-based composites, fiber content in vinyl ester composites, and impingement angle in epoxy composites. The morphological analysis exemplified the overarching wear-performance of epoxy-based composites reduced by the interaction of fibers with the contact mating-surface took place by fiber pull-out. Large wear debris was spotted in vinyl ester-based composites as revealed by the SEM analysis. These wear debris formed due to the detachment of sub polymeric material from base material due to low van der wall forces. In case of polyester composites, the examination revealed that main wear mechanism responsible for material removal is groove formation and micro-ploughing. The developed fiber-reinforced polymer sandwich composites are showing significant properties for orthopaedic, bone-fracture fixation applications. The present work can also be investigated for various other polymers such as PE, PC, PLA and PHB for further new possibilities of the composite for better performance. Fillers such as dolomite and marble dust powder can also be added in the composite to further enhance the erosive behaviour of the present composite.

## Figures and Tables

**Table 1:** Composition of fabricated composites.

S. No.	Designation	Compositions
1	E0	Epoxy + Glass fiber (5 wt%)
2	E1	Epoxy + glass fiber (5 wt%) + jute (2.5 wt%) + *Grewia optiva* (2.5 wt%)
3	E2	Epoxy + glass fiber (5 wt%) + jute (5 wt%) + *Grewia optiva* (5 wt%)
4	E3	Epoxy + glass fiber (5 wt%) + jute (7.5 wt%) + *Grewia optiva* (7.5 wt%)
5	V0	Vinyl ester + glass fiber (5 wt%)
6	V1	Vinyl ester + glass fiber (5 wt%) + jute (2.5 wt%) + *Grewia optiva* (2.5 wt%)
7	V2	Vinyl ester + glass fiber (5 wt%) + jute (5 wt%) + *Grewia optiva* (5 wt%)
8	V3	Vinyl ester + glass fiber (5 wt%) + jute (7.5 wt%) + *Grewia optiva* (7.5 wt%)
9	P0	Polyester + glass fiber (5 wt%)
10	P1	Polyester + glass fiber (5 wt%) + jute (2.5 wt%) + *Grewia optiva* (2.5 wt%)
11	P2	Polyester + glass fiber (5 wt%) + jute (5 wt%) + *Grewia optiva* (5 wt%)
12	P3	Polyester + glass fiber (5 wt%) + jute (7.5 wt%) + *Grewia optiva* (7.5 wt%)

**Table 2:** Control factors and respective levels.

Control Factors	Levels				
	I	II	III	IV	Units
Impact Velocity	30	40	50	60	m/s
Impingement angle	45	60	75	90	degree
Fiber content					
(For composites E_1_, E_2_, E_3_)	0	05	10	15	wt%
(For composites V_1_, V_2_, V_3_)	0	05	10	15	
(For composites P_1_, P_2_, P_3_)	0	05	10	15	

**Table 3:** Design-matrix array arrangement.

S. No.	Impact Velocity (m/s)	Natural Fiber (wt%)	Impingent Angle (Degree)
1	30	0	45
2	30	5	60
3	30	10	75
4	30	15	90
5	40	0	60
6	40	5	45
7	40	10	90
8	40	15	75
9	50	0	75
10	50	5	90
11	50	10	45
12	50	15	60
13	60	0	90
14	60	5	75
15	60	10	60
16	60	15	45

**Table 4:** List of work carried out for erosive wear using Taguchi experiment.

S. No.	Composition	Optimization Technique (Taguchi)	Control Factor with Corresponding Level					Highlights of Work	References
1	*Grewia optiva*–glass fiber–dolomite filler–epoxy	L_16_ orthogonal array	Impact velocity:	10	20	30	40	Highest influence on erosive wear was shown by impact velocity followed by dolomite content and erodent size. Lowest wear was obtained at impingent angle of 30°	[45]
			Dolomite content:	0	5	10	15		
			Impingement angle	30°	45°	60°	90°		
			Erodent size:	100	150	200	250		
2	Jute–SiC–epoxy	L_9_ orthogonal array	Impact velocity	32		44	58	Erosive wear increased with the increase in impact velocity, fiber content and impingement angle. Erodent size showed least effect on erosive wear.	[46]
			Impingement angle	30		60	90		
			Erodent size:	200		300	400		
			Fiber content	20		30	40		
3	Al_2_O_3_-glass fiber-polyester	L_27_ orthogonal array	Impact Velocity	43		54	65	At low impact velocity, the composite responded in a semi ductile manner while at high velocity, the composite responded in a ductile manner	[47]
			Filler content	0		10	20		
			Impingement angle	30		60	90		
			Stand-off distance	65		75	85		
			Erodent size	250		350	450		
4	AlN-glass fiber-epoxy	L_9_ orthogonal array	Impact Velocity	33		47	57	Most influential parameter in the analysis of erosive was impact velocity followed by temperature and filler content respectively.	[48]
			Filler content	5		10	15		
			Impingement angle	30		60	90		
			Temperature	50		75	100		
5	Bagasse fiber-epoxy	L_27_ orthogonal array	Impact Velocity	30		50	70	Around 80% influence of fiber weightage was observed in the erosive wear followed by 14% impingement angle and 4% impact velocity. Maximum erosive wear was observed at high fiber weightage of impingement angle of 60°	[49]
			Filler content	10		20	30		
			Impingement angle	30		60	90		
			Stand-off distance	65		75	85		
			Erodent size	250		350	450		
6	Needle punched Polyester fiber mat-epoxy	L_27_ orthogonal array	Impact Velocity	43		54	65	Erosive wear increased with the increase in impingement angle till 60° but as the impingement angle increased beyond 60°, the erosive wear decreased. Composite exhibited semi ductile erosive wear.	[50]
			Filler content	10		20	30		
			Impingement angle	30		60	90		
			Stand-off distance	65		75	85		
			Erodent size	250		350	450		
7	Palm leaf fiber-epoxy-palm leaf powder	L_16_ orthogonal array	Impact Velocity	40	50	60	70	Composite with 15% palm leaf fiber at 60° impingement angle and impact velocity of 80 m/s showed the highest wear erosion resistance.	[51]
			Filler content	0	5	10	15		
			Impingement angle	45	60	75	90		
			Erodent size	40	60	80	100		
8	E glass fiber-SiC-epoxy	L_27_ orthogonal array	Impact Velocity	32		45	58	A significant reduction in erosive wear was observed by the addition of SiC in glass fiber composite. Maximum wear erosion has occurred at 60°. Composite transform in brittle structure with the incorporation of SiC.	[52]
			SiC content	0		10	20		
			Impingement angle	30		60	90		
			Stand-off distance	120		180	240		
			Erodent size	300		500	800		
9	Himalayan agave fiber-polyester	L_16_ orthogonal array	Sliding Velocity	1.5	2.5	3.5	4.5	Composite with fiber of 5 mm length exhibited the highest erosive wear resistance. Longer fiber (7 mm) reinforced composite exhibited greater erosive wear due to fiber fracture and surface damage. Optimum parameters efficient erosive wear resistance were reported as sliding velocity: 15 m/s, normal load: 20 N, fiber length: 5 mm, and sliding distance: 1500 m.	[53]
			Fiber Length	0	3	5	7		
			Sliding Distance	1000	2000	3000	4000		
			Normal Load	10	15	20	25		
10	Glass fiber-fly ash-polyester	L_27_ orthogonal array	Impact Velocity	32		45	58	The highest erosive wear of composite occurred at 60° of impingement angle and showed semi ductile behavior. Fly ash content has the highest influence on erosive wear in terms of influencing factors, followed by impingement angle and erodent size. Impact velocity has minimum impact on erosive wear, as reported in the study.	[54]
			Fly ash Content	0		10	20		
			Impingement angle	45		60	90		
			Stand-off distance	120		180	240		
			Erodent size	300		500	800		

Note: Impact velocity: (m/s), erodent size: (mm), impingement angle: (degree), stand-off distance: (mm), normal load: (N), sliding distance: (m), fiber/filler content: (weight percentage), fiber length: (mm).

**Table 5:** Mechanical properties of composites.

Sample	Mechanical Properties			
	Tensile Strength (MPa)	Flexural Strength (MPa)	Impact Strength (J)	Hardness (HRL)
E0	38	22	1.2	52
E1	54	38	1.4	68
E2	68	42	1.68	76
E3	72	36	2.1	57
V0	32	24	1.1	38
V1	47	42	1.2	48
V2	62	48	1.5	54
V3	69	38	1.9	42
P0	28	18	0.9	44
P1	42	28	1.05	56
P2	56	35	1.26	64
P3	60	31	1.71	51

**Table 6:** Erosive wear and corresponding S/N ratio of the composites.

S. No.	Erosive Wear of Epoxy (mg/kg)	S/N Ratios	Erosive Wear of Vinyl Ester (mg/kg)	S/N Ratios	Erosive Wear of Polyester (mg/kg)	S/N Ratios
1	298.96	−49.5123	304.67	−49.6766	309.88	−49.8239
2	218.47	−46.7878	221.08	−46.8910	232.10	−47.3135
3	219.06	−46.8113	236.11	−47.4623	228.07	−47.1614
4	209.65	−46.4299	305.44	−49.6985	253.45	−48.0778
5	338.75	−50.5976	445.21	−52.9713	318.85	−50.0717
6	260.37	−48.3118	278.021	−48.8816	297.92	−49.4820
7	309.44	−49.8115	211.67	−46.5132	247.81	−47.8824
8	311.23	−49.8616	309.37	−49.8096	384.53	−51.6986
9	461.64	−53.2861	361.09	−51.1523	506.43	−54.0904
10	416.86	−52.3998	398.56	−52.0099	434.67	−52.7632
11	306.99	−49.7425	380.88	−51.6158	371.09	−51.3896
12	386.25	−51.7374	432.01	−52.7099	489.56	−53.7961
13	527.65	−54.4469	550.84	−54.8205	588.19	−55.3904
14	520.97	−54.3363	554.41	−54.8766	566.71	−55.0672
15	364.06	−51.2235	381.56	−51.6313	408.34	−52.2204
16	411.85	−52.2948	415.51	−52.3716	421.01	−52.4858

**Table 7:** Response table for composites.

Levels	Epoxy			Vinyl Ester			Polyester		
	Impact Velocity (m/s)	Fiber Content (wt%)	Impingement Angle (Degree)	Impact Velocity (m/s)	Fiber Content (wt%)	Impingement Angle (Degree)	Impact Velocity (m/s)	Fiber Content (wt%)	Impingement Angle (Degree)
1	−47.39	−51.96	−49.97	−48.43	−52.16	−50.64	−48.09	−52.34	−50.8
2	−49.65	−50.46	−50.09	−49.54	−50.66	−51.05	−49.78	−51.16	−50.58
3	−51.79	−49.40	−51.07	−51.87	−49.31	−50.83	−53.01	−49.66	−50
4	−53.08	−50.08	−50.77	−53.43	−51.15	−50.76	−53.79	−51.51	−51
Delta	5.69	2.56	1.11	4.99	2.85	0.41	5.7	2.68	1.21
Rank	1	2	3	1	2	3	1	2	3

**Table 8:** Analysis of variance for SNRA2, using adjusted SS for tests epoxy-based composites.

Source	DF	Seq SS	Adj SS	Adj MS	F-Value	*p*-Value
Impact Velocity (m/s)	3	74.915	74.915	24.972	26.43	0.001
Fiber content (wt%)	3	14.098	14.098	4.699	4.97	0.046
Impingement angle (Degree)	3	3.43	3.43	1.143	1.21	0.384
Error	6	5.67	5.67	0.945		
Total	15	98.113				

**Table 9:** Analysis of Variance for SNRA2, using adjusted SS for tests vinylester based composites.

Source	DF	Seq SS	Adj SS	Adj MS	F-Value	*p*-Value
Impact velocity (m/s)	3	60.893	60.893	20.298	5.97	0.031
Fiber content (wt%)	3	16.829	16.829	5.61	1.65	0.275
Impingement angle (Degree)	3	0.362	0.362	0.121	0.04	0.99
Error	6	20.405	20.405	3.401		
Total	15	98.489				

**Table 10:** Analysis of variance for SNRA2, using adjusted SS for tests polyester.

Source	DF	Seq SS	Adj SS	Adj MS	F-Value	*p*-Value
Impact Velocity (m/s)	3	86.549	86.549	28.85	29.58	0.001
Fiber content (wt%)	3	15.069	15.069	5.023	5.15	0.043
Impingement angle (Degree)	3	3.835	3.835	1.278	1.31	0.355
Error	6	5.853	5.853	0.975		
Total	15	111.305				

## Data Availability

The data presented in this study are available on request from the corresponding author.
